# Inflammation of the Gonad in Prepubertal Healthy Children. Epidemiology, Etiology, and Management

**DOI:** 10.1100/tsw.2006.200

**Published:** 2006-08-31

**Authors:** Sarel Halachmi

**Affiliations:** Department of Urology, Rambam Medical Center, Faculty of Medicine Technion, Israeli Institute of Technology, Haifa, Israel

**Keywords:** gonadal inflammation, prepubertal children, prevalence, etiology, management, Israel

## Abstract

The prevalence, etiology, and proper management of acute gonadal inflammation in prepubertal children are still controversial, with some reports defining it as rare, while others have found it more prevalent. So far, there is no consensus on imaging studies or standard follow-up procedures. In the minority of the children, the inflammation is related to congenital genitourinary malformation and bacterial infection. The majority of children with gonadal inflammation are healthy and do not have any underlying malformations; in this group, the etiology is related to viral infection or torsion of the gonad appendix. Management is directed towards the etiology. Hence, when bacterial inflammation is suspected, antibiotics should be given and full evaluation of the urinary tract system should be performed. For patients with negative medical history, absence of fever, and normal urinalysis, the diagnosis of bacterial inflammation is very unlikely, and there is neither justification for antimicrobial antibiotic therapy nor for any further urinary tract imaging. Caution should be taken with nonverbal children and infants, or patients with any abnormal parameter. For these patients, we recommend initial management as for bacterial urinary tract infection, until urine cultures results are obtained. This paper provides a comprehensive review with the related medical literature.

### INTRODUCTION

The prevalence, etiology, clinical course, and management of gonadal inflammation (GI) in prepubertal children are not yet clear. The relevant data in the medical literature are few and sometimes inconsistent. For instance, there are studies that define the disease as rare and others as more prevalent. Despite the fact that the etiology is considered “enigmatic”, an antimicrobial course is still recommended[1]. There is no consensus regarding imaging studies or follow-up standards. This review will try to give an organized view on this subject according to the available medical literature.

### PREVALENCE OF GONADAL INFLAMMATION IN PREPUBERTAL CHILDREN

Gonadal inflammation in prepubertal children was thought to be a rare phenomenon. In their chapter dealing with inflammatory disease of the genitourinary tract, Kennedy and Shortliffe[1] considered this disease as a relatively rare problem in children. However, the data presented in several other studies should change this statement. Lewis et al.[2] retrospectively analyzed the distribution of the etiologies for acute scrotal pain in children admitted to the emergency room over a period of 2 years. In this group, 192/238 (81%) of the children had “inflammatory” process: 109/238 (46%) had torsion of the appendix testis and 83/238 (35%) had inflammation of the gonad. For comparison only, 16% had torsion of the testis. Klin et al.[3] showed similar results in their study. Among the 65 children who were referred within 5 years due to acute scrotal pain, 42 (65%) had “epididymitis” and 5 (8%) had torsion of the appendix testis; 12 patients (18%) had torsion of the testis. Ben Chaim et al.[4] described the surgical findings in 70 children who underwent exploration of the scrotum due to acute pain. In 33 children (47%), torsion of the appendages was found. Testicular torsion was diagnosed in 24 children (34%). Halachmi et al.[5] reported about 193 patients with GI in a period of 5 years. McAndrew et al.[6] showed that GI and torsion of the gonadal appendages were a frequent event with a study over 14 months of 100 children admitted due to acute scrotal pain. Among the 100 consecutive children studied, 70 had torsion of the appendix and 12 had inflammation. There were, overall, 8,932 admissions in the same period of time giving an overall incidence of 0.8% for GI patients. Somekh et al.[7] estimated the prevalence of GI in children to be 1.2/1,000 children/year. The data from the above-mentioned research proved that inflammation of the gonad in prepubertal children is indeed not a rare event.

### ETIOLOGY FOR GONADAL INFLAMMATION IN PREPUBERTAL CHILDREN

#### Congenital Malformations and Bacterial Infection

Relatively old studies targeted congenital urological genital and neurourological malformations as the cause for GI. Siegel et al.[8] described a group of 17 prepubertal children hospitalized for GI. Eight (47%) of them had congenital malformation of the urinary tract; 6/8 (75%) had positive cultures for coliform bacteria. It is also known that children with anorectal malformations have associated urological problems, including bacterial inflammation of the testis and the epididymis[9,10,11]. Bukowski et al.[12] postulated that dysfunctional voiding was associated with GI. The author performed urodynamic studies in 56 boys with epididymitis; 31 of them were prepubertal. Ten out of the 31 (32%) had the diagnosis of dysfunctional voiding with the possibility of vesicovasal reflux of urine causing chemical irritation of the gonad. Although cystograms were performed in some of the patients, none demonstrated reflux towards the gonad. The impression from this study was that the gonadal inflammation was related to bacteruria and urinary tract infection due to the voiding dysfunction. In summary, congenital anatomical and neurological malformations of the genitourinary system lead to bacterial infection of the urinary tract that may involve the gonad.

#### Nonbacterial Causes

From our clinical experience, most of the children presenting with GI at the emergency department are healthy and do not have any congenital malformations. Our clinical impression is supported by various studies. Halachmi et al.[5] analyzed retrospectively the clinical and laboratory data of 193 consecutive children admitted to the emergency department for scrotal pain and diagnosed with GI. All the children in this study group had urine cultures; 182/193 (94.3%) cultures were negative and 11/193 (5.6%) positive. Among the negative culture group, only three children (1/6%) had symptoms related to urinary tract infection, such as dysuria, urgency, or frequency. In all 182 children with negative cultures, medical history, physical examination (beside local scrotal findings), and body temperature were normal. Scrotal ultrasound examination showed hyperemia in 80%, torsion of the gonadal appendages in 5%, and normal in 15%. In the remaining 11 children with positive urinary cultures, 73% had symptoms related to urinary tract infection; 73% had positive medical history related to urological or neurourological malformation. The study concluded that most of the patients did not have bacterial GI. Patients with nonbacterial GI were usually prepubertal, healthy children without positive history for urological disease or anomaly. The presenting symptom was scrotal pain and swelling, usually without urinary symptoms. Clinical presentation in addition to negative medical history, absence of fever, normal urinalysis, and sterile culture makes the diagnosis of bacterial GI very unlikely.

### Viral Infection

Somekh at al.[7] presented similar results in their study among 44 children with GI; only one (2.7%) had positive urine culture. While looking for the etiology causing the inflammation, they found serological evidence for recent viral infection in 20% of the patients. All 43 children were treated conservatively and recovered spontaneously. The authors suggested that epididymitis in boys was not rare and mostly an inflammatory phenomenon (presumably postviral infectious) with a benign course. Greenfield[13] supported the etiology of viral infection as the cause for GI in a review of nine cases of epididymitis due to *Haemophilus influenza* type b infection. Tassar et al.[14] described a rare case of epididymitis related to acute hepatitis B infection and orchitis as a well-described complication of mumps infection usually in postpubertal boys[15].

### Torsion of the Gonadal Appendages

Ben Chaim et al.[4] analyzed the surgical findings in 70 children undergoing exploration for acute scrotal pain. In 33 (47%) of the children, torsion of the gonadal appendix was found as the cause for the inflammation. Hegarty et al.[16] published similar results. Exploration of 100 consecutive boys with scrotal pain yielded a rate of 32% of gonadal appendage torsion, and 20% inflammation. McAndrew et al.[6] showed a 70% rate of torsion of the appendix testis in 100 consecutive patients admitted for scrotal pain during a period of 14 months. In summary, nonbacterial inflammation of the gonad was related to gonadal appendage torsion and viral infection.

## IMAGING STUDIES

### Imaging of the Scrotum

Ultrasound imaging of the scrotum has become one of the major tools in the evaluation of patients with acute scrotum due to its superior resolution and sensitive Doppler capabilities[17]. Routine scrotal exploration is no longer recommended for all children with an acute scrotum in whom Doppler ultrasound demonstrated blood flow to the testis[18]. The testis, epididymis, and gonadal appendages are well visualized during scrotal sonography[19]. Sellars and Sidhu[20] and Strauss et al.[21] described the sonographic appearance of torsed gonadal appendix; the appendix was enlarged, homogeneously echogenic, seen medial or posterior to the head of the epididymis. The situation may be accompanied by hydrocele and thickening of the scrotal wall, enlargement of the head of the epididymis, and increased testicular blood flow. Monga et al.[22] described similar findings, i.e., demonstration of a pedunculated mass with a central hypoechoic area at the superior aspect of the testis, supporting the diagnosis of torsed gonadal appendix. Halachmi et al.[23] repeated scrotal ultrasound in a child with clear sonographic diagnosis of gonadal appendix torsion. Within 24–36 h following the first ultrasound, the whole epididymis and then the testis became inflamed, showing nonspecific inflammation masking the ability to identify the torsed appendix. Hence, children with torsed gonadal appendix presenting a few days after symptoms started may not be diagnosed sonographicaly, probably explaining the nonsignificant sonographic findings in many cases.

#### Further Imaging of the Urinary Tract System

Proven bacterial urinary tract infection mandates full evaluation of the urinary tract including invasive studies, such as voiding cystourethrography (VCUG). Siegel et al.[8] demonstrated diagnosis of various urogenital anomalies while performing VCUG in children with bacterial GI. For nonbacterial inflammation, there is no need for any further imaging studies. Halachmi et al.[5] showed that in 31 patients with nonbacterial inflammation of the gonad, further imaging studies, such as renal ultrasound, IVP, and VCUG, did not show any pathology. Cappele et al.[24] performed ultrasound and VCUG in 38 patients with acute epididymitis; 12 (31%) bacterial infection, 26 (79%) nonbacterial. Only seven patients (18%) had any urinary tract anomaly, some of which were not directly associated with the GI, such as renal malrotation and ureteropelvic junction obstruction. The remaining anomalies included vesicoureteral reflux, obstructing ureterocele, seminal vesicle reflux, and utricular reflux. The patient with the ureterocele was the only one to undergo corrective surgery. The authors concluded that with no previously known uropathies and no bacteriuria, further imaging investigation of the urinary tract should be performed only after the second episode.

### MANAGEMENT

Bacterial urinary tract infection should be highly suspect in patients with positive history for urological and neurourological congenital malformations, and patients presenting with symptoms such as dysuria, frequency, and urgency. Elevated body temperature, accompanied by general symptoms such as vomiting, apathy, malaise, and abnormal urinalysis, should also direct the physician towards bacterial infection. Care should be taken with young infants and nonverbal children, or persons with intellectual disability. In this group, it is hard to obtain accurate medical history and symptoms are not as pathognomonic as in adults[5]. For patients with a high suspicion for bacterial urinary tract infection, proper management includes the administration of antibiotics directed towards gram negative bacteria until final culture results are obtained.

For patients in whom bacterial infection is unlikely, i.e., there is negative medical history, no dysuria, normal body temperature, and clear urinalysis, conservative management including rest, scrotal elevation, and nonsteroidal anti-inflammatory drugs (NSAIDs) is the proper strategy. Somekh et al.[7] showed spontaneous resolution of the inflammation within a week without any treatment beside NSAIDs. Proven torsion of the gonadal appendages is also amenable for the same conservative management[24]. Cappele et al.[24], examining patients with nonbacterial epididymitis treated conservatively 1 month after the acute event, showed complete resolution of the inflammatory process without any effect on the gonad size and consistency.

Despite the fact that patients with nonbacterial GI do not present with any typical symptoms of urinary tract infection, there is a nonjustified tendency for the administration of antibiotics. Halachmi et al.[5] showed that an antibiotic course was given to 142/169 (78%) children with nonbacterial GI.

### CONCLUSIONS

Inflammation of the gonad in prepubertal children is not a rare event. In the minority of the children, the inflammation is related to congenital genitourinary malformation and bacterial infection. The majority of children with GI are healthy and do not have any underlying malformations; in this group, the etiology is related to viral infection or torsion of the gonad appendix. Management is directed towards the etiology. Hence, when bacterial inflammation is suspected, antibiotics should be given and full evaluation of the urinary tract system should be performed. For patients with negative medical history, absence of fever, and normal urinalysis, the diagnosis of bacterial inflammation is very unlikely, and there is neither justification for antimicrobial antibiotic therapy nor for any further urinary tract imaging. Caution should be taken with nonverbal children and infants, or patients with any abnormal parameter. For these patients, we recommend initial management as for bacterial urinary tract infection, until urine cultures results are obtained.
